# Supplementary material for: Excitatory rubral cells encode the acquisition of novel complex motor tasks
Source: Nat Commun. 2019 May 21;10:2241. doi: 10.1038/s41467-019-10223-y (PMC6529416; doi:10.1038/s41467-019-10223-y)
Supplement: Supplementary file 1 — Supplementary information [file 41467_2019_10223_MOESM1_ESM.pdf]

Supplementary Information

Excitatory rubral cells encode the acquisition of novel complex motor tasks

Rizzi et al.

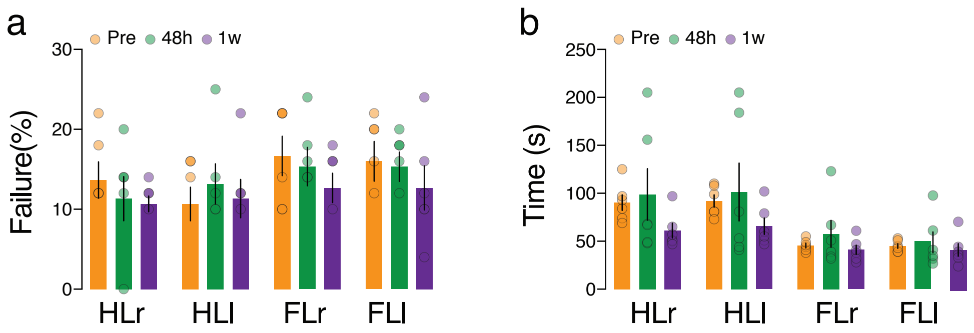

Supplementary Figure 1: There is no performance adaptation present in the SGT. a, Failure rate. b, Completion time. (n=6 mice). Data are reported as mean and SEM.

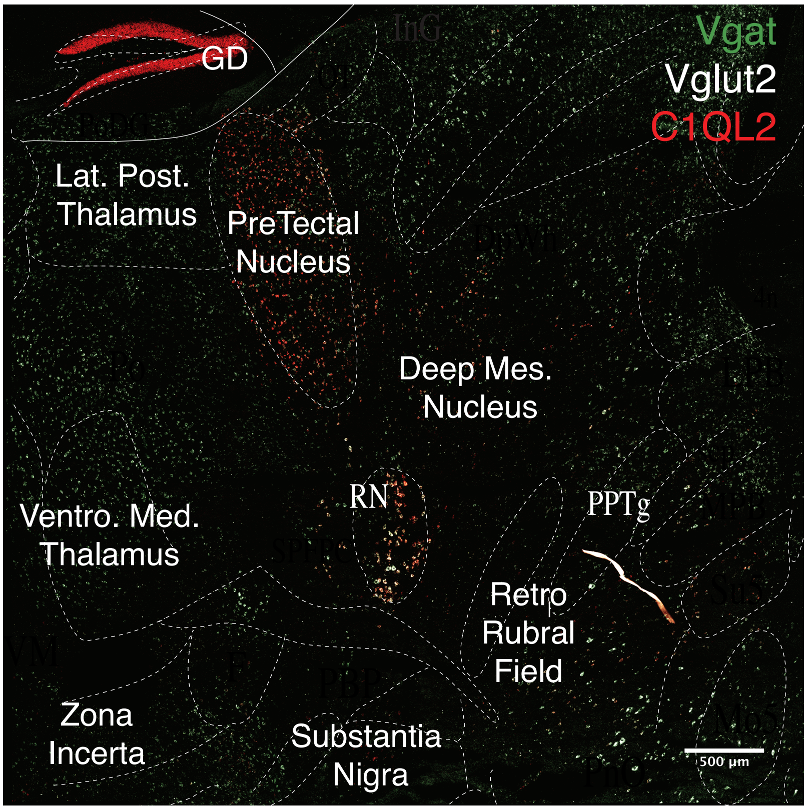

Supplementary Figure 2: Localization and co-expression of Vgat, Vglut2 and C1QL2. Sagittal section containing the RN that was processed for Vgat, Vglut2 and C1QL2 mRNA labelling. Scale bar 500µm (n=3 mice).

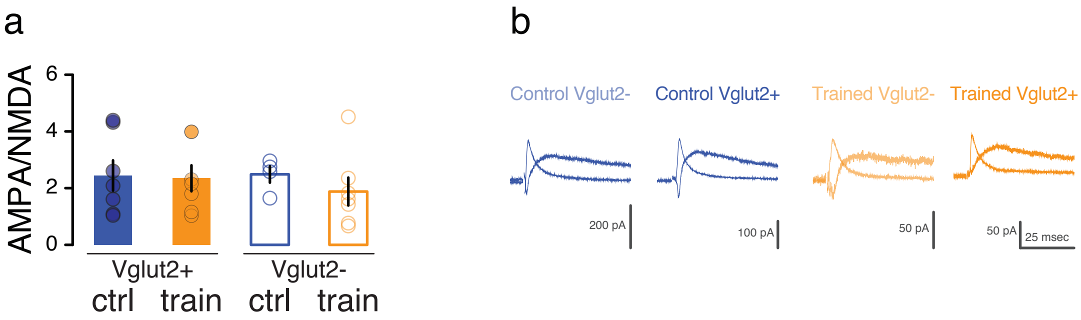

Supplementary Figure 3: SPRT training-dependent plasticity does not modify the AMPA/NMDAR ratio. a, AMPA/NMDA ratio. b, Example traces. (n=5-7 cells/group). Data are reported as mean and SEM. (Ctrl: control, train: trained).

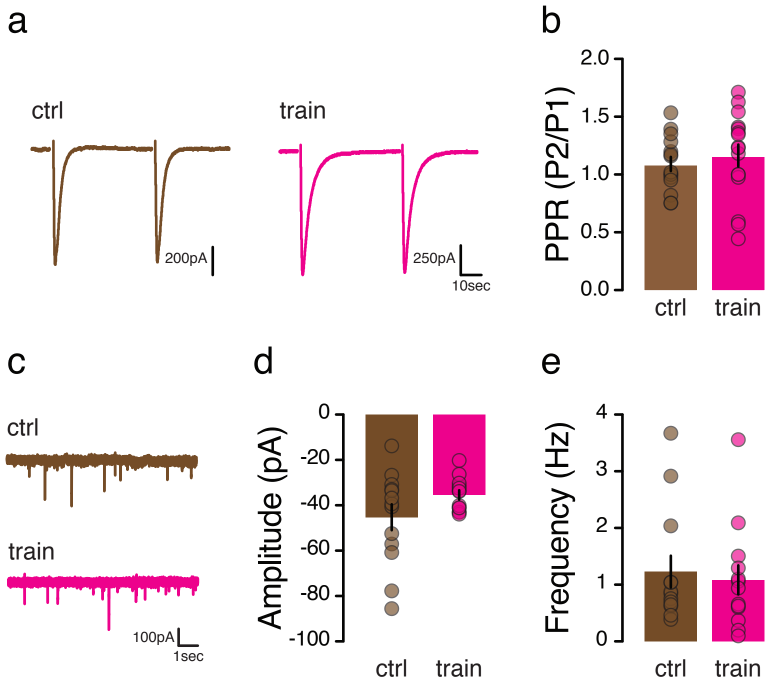

Supplementary Figure 4: SPRT training does not modify inhibitory transmission. a, IPSC example traces. b, Paired-pulse ratio (n=15-17 cells/group). c, mIPSC example traces. d, mIPSC amplitude. e, mIPSC frequency. (n=13 cells/group). Data are reported as mean and SEM. Ctrl: control, train: trained.

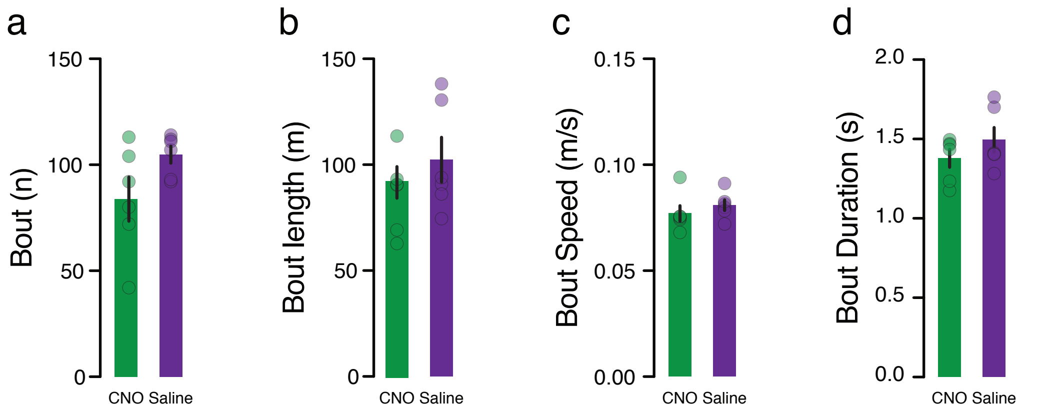

Supplementary Figure 5: Chemogenetic inhibition of Vglut2-positive cells does not affect spontaneous locomotion. a, Bout number. b, Length. c, Speed. d, Duration. (n=6 mice). Data are reported as mean and SEM.
