# Supplementary material for: Excitatory rubral cells encode the acquisition of novel complex motor tasks
Source: Nat Commun. 2019 May 21;10:2241. doi: 10.1038/s41467-019-10223-y (PMC6529416; doi:10.1038/s41467-019-10223-y)
Supplement: Supplementary file 2 — Description of Additional Supplementary Files [file 41467_2019_10223_MOESM2_ESM.docx]

Description of Additional Supplementary Files

**Supplementary Movie 1:** RN light ablation spares spontaneous locomotion. Example of a mouse exploring an open arena before and after blue light lesion of the RN.

**Supplementary Movie 2:** RN light ablation impairs grasping skills. Performance on the suspended elevated grid test is decreased after light lesion of the RN.

**Supplementary Movie 3:** RN light ablation impairs reaching skills. Performance in the single pellet reaching task is decreased after light lesion of the RN.
