# Supplementary material for: Excitatory rubral cells encode the acquisition of novel complex motor tasks
Source: Nat Commun. 2019 May 21;10:2241. doi: 10.1038/s41467-019-10223-y (PMC6529416; doi:10.1038/s41467-019-10223-y)
Supplement: Supplementary file 7 — Reporting Summary [file 41467_2019_10223_MOESM7_ESM.pdf]

nature research

Corresponding author(s): Kelly R Tan
kelly.tan@unibas.ch

Last updated by author(s): Apr 2, 20019

Reporting Summary

Nature Research wishes to improve the reproducibility of the work that we publish. This form provides structure for consistency and transparency in reporting. For further information on Nature Research policies, see Authors & Referees and the Editorial Policy Checklist.

Statistics

For all statistical analyses, confirm that the following items are present in the figure legend, table legend, main text, or Methods section.

n/a	Confirmed	
☐	☒	The exact sample size (*n*) for each experimental group/condition, given as a discrete number and unit of measurement
☐	☒	A statement on whether measurements were taken from distinct samples or whether the same sample was measured repeatedly
☐	☒	The statistical test(s) used AND whether they are one- or two-sided<br>*Only common tests should be described solely by name; describe more complex techniques in the Methods section.*
☐	☒	A description of all covariates tested
☐	☒	A description of any assumptions or corrections, such as tests of normality and adjustment for multiple comparisons
☐	☒	A full description of the statistical parameters including central tendency (e.g. means) or other basic estimates (e.g. regression coefficient) AND variation (e.g. standard deviation) or associated estimates of uncertainty (e.g. confidence intervals)
☐	☒	For null hypothesis testing, the test statistic (e.g. *F*, *t*, *r*) with confidence intervals, effect sizes, degrees of freedom and *P* value noted<br>*Give P values as exact values whenever suitable.*
☒	☐	For Bayesian analysis, information on the choice of priors and Markov chain Monte Carlo settings
☒	☐	For hierarchical and complex designs, identification of the appropriate level for tests and full reporting of outcomes
☒	☐	Estimates of effect sizes (e.g. Cohen's *d*, Pearson's *r*), indicating how they were calculated

Our web collection on statistics for biologists contains articles on many of the points above.

Software and code

Policy information about availability of computer code

Data collection	Part of the behavioral experiments were conducted using AnyMaze 5.14(Stoelting) .<br>Electrophysiological recordings were performed using Signal 6.04 (CED).
Data analysis	Data analysis was performed using Matlab r2017b (Mathworks).

For manuscripts utilizing custom algorithms or software that are central to the research but not yet described in published literature, software must be made available to editors/reviewers. We strongly encourage code deposition in a community repository (e.g. GitHub). See the Nature Research guidelines for submitting code & software for further information.

Data

Policy information about availability of data

All manuscripts must include a data availability statement. This statement should provide the following information, where applicable:
- Accession codes, unique identifiers, or web links for publicly available datasets
- A list of figures that have associated raw data
- A description of any restrictions on data availability

The data sets generated during and/or analyzed during the current study are available from the corresponding author on reasonable request.

Field-specific reporting

Please select the one below that is the best fit for your research. If you are not sure, read the appropriate sections before making your selection.

☒ Life sciences ☐ Behavioural & social sciences ☐ Ecological, evolutionary & environmental sciences

For a reference copy of the document with all sections, see nature.com/documents/nr-reporting-summary-flat.pdf

Life sciences study design

All studies must disclose on these points even when the disclosure is negative.

Sample size	All sample sizes were coherent with standard practices in our field and multiple publications in recognized scientific journals. The sufficiency of the sample sizes was estimated with post-hoc power analysis.
Data exclusions	The only exclusion criterion was post-hoc validation of the viral injection and fiber placement where applicable.
Replication	All attempts of replication were successful and reflected in the ns reported.
Randomization	Mice were randomly allocated in the respective groups based on genotype where applicable.
Blinding	When possible, (electrophysiology and DREADD experiments) the experimenter acquiring the data was blind to the experimental condition.

Reporting for specific materials, systems and methods

We require information from authors about some types of materials, experimental systems and methods used in many studies. Here, indicate whether each material, system or method listed is relevant to your study. If you are not sure if a list item applies to your research, read the appropriate section before selecting a response.

Materials & experimental systems

n/a	Involved in the study	
☐	☒	Antibodies
☒	☐	Eukaryotic cell lines
☒	☐	Palaeontology
☐	☒	Animals and other organisms
☒	☐	Human research participants
☒	☐	Clinical data

Methods

n/a	Involved in the study	
☒	☐	ChIP-seq
☒	☐	Flow cytometry
☒	☐	MRI-based neuroimaging

Antibodies

Antibodies used	NeuroTrace™ 500/525 Green Fluorescent Nissl Stain - Solution in DMSO (Thermofisher).
Validation	The antibody has been validated and the relevant information can be found under Catalog number: N21480.

Animals and other organisms

Policy information about studies involving animals; ARRIVE guidelines recommended for reporting animal research

Laboratory animals	(Jax Laboratories) STOCK Slc17a6<tm2(cre)Lowl>/J Adult Male & Female ; (Janvier) C57BL/6JRj Adult Male & Female
Wild animals	N/A
Field-collected samples	N/A
Ethics oversight	All animals and procedures were approved by the Institutional Animal Care office of the University of Basel and the Cantonal Veterinary office.

Note that full information on the approval of the study protocol must also be provided in the manuscript.
